# The Class III Peroxidase Gene Family in *Populus simonii*: Genome-Wide Identification, Classification, Gene Expression and Functional Analysis

**DOI:** 10.3390/antiox14050602

**Published:** 2025-05-16

**Authors:** Lu Han, Yishuang Ren, Xinru Bi, Guowei Yao, Jinwang Zhang, Hongtao Yuan, Xiaoyu Xie, Junbo Chen, Yunchang Zhang, Sitong Du, Wanying Chen, Kewei Cai, Xiyang Zhao

**Affiliations:** 1 Jilin Provincial Key Laboratory of Tree and Grass Genetics and Breeding, College of Forestry and Grassland Science, Jilin Agricultural University, Changchun 130118, China; 20230611@mails.jlau.edu.cn (L.H.); renyishuang@mails.jlau.edu.cn (Y.R.); bixinru@mails.jlau.edu.cn (X.B.); yaoguowei@mails.jlau.edu.cn (G.Y.); 20230599@mails.jlau.edu.cn (X.X.); chenjunbo@mails.jlau.edu.cn (J.C.); zhangyunchang@mails.jlau.edu.cn (Y.Z.); dusitong@mails.jlau.edu.cn (S.D.); chenwanying@mails.jlau.edu.cn (W.C.); 2 Tongliao Forestry and Grassland Science Research Institute, Tongliao 028000, China19997161499@163.com (H.Y.)

**Keywords:** *Populus simonii*, class III peroxidase (POD), expression pattern, protein–protein interaction

## Abstract

Class III peroxidases are plant-specific enzymes that play indispensable roles in catalyzing oxidative–reductive reactions, which are integral to numerous biochemical processes in plants. In this study, we identified 69 members of the class III peroxidase (*POD*) gene family in the *Populus simonii* genome and classified them into four subfamilies based on phylogenetic analysis. Chromosomal localization revealed that these *PsPOD* genes are unevenly distributed across 19 chromosomes, with chromosomes 3 and 7 harboring the highest densities. Conserved domain and motif analyses demonstrated that all *PsPOD* proteins contain the characteristic peroxidase domain and share highly conserved motif structures. Cis-acting element analysis of promoter regions revealed the presence of numerous regulatory elements associated with light responsiveness, phytohormone signaling, stress responses, and plant growth and development. Transcriptome data showed that the expression of *PsPOD* genes varies significantly across different tissues and organs and under various stress conditions, suggesting their involvement in both developmental processes and abiotic stress responses. These findings were further validated by qRT-PCR analysis of selected *PsPOD* genes. Notably, *PsPOD45*, *PsPOD69*, *PsPOD33*, and *PsPOD64* were identified as central hub genes in the protein–protein interaction network, making them promising candidates for further functional characterization. Overall, this study provides a comprehensive overview of the *PsPOD* gene family in *P. simonii*, laying a solid foundation for future functional studies and offering valuable insights for comparative research in other plant species.

## 1. Introduction

*Populus simonii*, a perennial deciduous tree in the *Populus* genus (family Salicaceae), is one of China’s most important native tree species. It plays a key role in the shelterbelt systems of northeastern and northwestern China, contributing significantly to windbreaks, sand fixation, slope stabilization, and soil-water conservation [1]. These ecological functions are especially valuable in arid and semi-arid regions, where *P. simonii* helps combat soil erosion and supports ecosystem rehabilitation [2]. In addition to its ecological utility, *P. simonii* exhibits strong drought resistance, cold tolerance, and high biomass productivity. It also demonstrates outstanding hybrid vigor and natural regeneration capacity, making it one of the earliest tree species in China to undergo artificial selection and genetic improvement [3]. Particularly during the afforestation campaigns of 1950–1969, it was regarded as a “pioneering species,” playing a crucial role in forestry ecological engineering and the regional economy of northern China [4]. Today, *P. simonii* remains foundational to artificial poplar plantations in desert and arid regions. With increasing global attention on stress adaptation and horticultural traits in poplars, *P. simonii* is increasingly recognized as a valuable genetic resource for breeding and improvement programs.

Peroxidase is a type of enzyme that is widely present in organisms, mainly catalyzing the redox reaction between hydrogen peroxide, which acts as an electron acceptor, and a variety of electron donors [5,6]. This process plays a crucial role in protecting cells from oxidative stress damage. When there is an imbalance in the oxidation–reduction state within cells, which could potentially harm the cells, the activity of peroxidase adjusts accordingly, maintaining the cell’s internal oxidation–reduction balance by decomposing peroxides [7]. This helps prevent oxidative damage such as lipid peroxidation, protein injury, and DNA damage, thereby maintaining the normal function of cells. Therefore, peroxidase plays an important role in preventing cell damage caused by oxidative stress [8]. Based on their protein sequences and structural features, peroxidases can be divided into non-heme peroxidases and heme peroxidases. The heme peroxidases are mainly divided into animal heme peroxidases and non-animal heme peroxidases [9]. According to their amino acid sequences and catalytic properties, non-animal heme peroxidases can be further subdivided into three categories: Class I peroxidases are intracellular, including yeast cytochrome c peroxidase, ascorbic acid peroxidase, and catalase-peroxidase sourced from bacteria; Class II peroxidases are extracellular, sourced from fungi, including various manganese peroxidases and lignin peroxidases produced by fungi [10,11]; Class III peroxidases are plant-specific redox enzymes, i.e., secretory peroxidases derived from higher plants that are involved in various physiological processes. Current research indicates that Class III peroxidases primarily serve two main functions in plants. On one hand, they are associated with normal morphogenesis and development, playing a crucial role in plant growth and developmental processes [12]. On the other hand, they contribute to plant stress resistance, including drought, cold, salt, and disease tolerance, making them a key component of the plant defense system [13]. However, there is an inconsistency in the naming conventions of Class III peroxidases, with abbreviations such as POX [14,15], POD [16,17], PRX [18], and GPX [19] being used interchangeably. In this paper, for the sake of brevity, we will refer to Class III peroxidase as POD.

Peroxidases (PODs) play crucial roles in diverse plant processes, including growth, development, stress responses, and lignin biosynthesis. Over the years, systematic studies on *POD* gene families have been conducted in many plant species, revealing their functions in maintaining plant homeostasis and coping with abiotic stress. For instance, in *Populus trichocarpa*, *Betula platyphylla*, and *Arabidopsis thaliana*, researchers have characterized *POD* gene families and elucidated their expression patterns in various tissues and under stress conditions [18,20,21]. Similarly, in crops such as maize, cassava, and fruits like lychee and Chinese pear, researchers have identified *POD* gene members and provided valuable insights into their roles in stress tolerance and secondary metabolism [22,23,24,25]. However, despite these advances, little is known about the *POD* gene family in *P. simonii*. Given the ecological and economic importance of *P. simonii* as a tree species with remarkable adaptability to arid and semi-arid environments, a comprehensive analysis of its *POD* gene family is warranted.

In this study, we conducted a comprehensive analysis of the class III peroxidase (*POD*) gene family in *P. simonii*. This analysis encompassed phylogenetic relationships, gene synteny, conserved functional domain characteristics, and the identification of cis-acting regulatory elements. Furthermore, we investigated the expression profiles of these *POD* genes across six distinct organs or tissues and their transcriptional responses to three abiotic stress conditions. To validate these findings, we randomly selected twelve *POD* genes for quantitative real-time PCR (qRT-PCR) analysis. This study aims to investigate the evolutionary expansion and adaptive characteristics of *POD* genes in *P. simonii*, providing insights into the molecular mechanisms underlying its ecological adaptability.

## 2. Materials and Methods

### 2.1. Plant Materials and Sample Collections

The experimental materials consisted of six-year-old clonal *P. simonii* trees cultivated under standardized conditions at the Tongliao Forestry and Grassland Science Research Institute, Inner Mongolia, China (44°01′05.300″ N, 121°59′03.300″ E). To ensure genetic uniformity, all specimens were vegetatively propagated from a single elite genotype selected for superior drought tolerance. During the active growth season (June 2022), samples were systematically collected from six distinct organs or tissues: apical buds (NT), axillary buds (NB), leaves (NL), one-year-old stems (NS), phloem (NP), and roots (NR). For each material type, three biological replicates were collected from separate individuals (n = 3). Immediately after excision, samples were flash-frozen in liquid nitrogen and stored at −80 °C until RNA extraction to preserve transcriptomic integrity [26]. Additionally, uniform one-year-old potted *P. simonii* plantlets were subjected to three abiotic stress treatments, including heat stress (plants were exposed to 42 °C for 6 h in a growth chamber), cold stress (plants were maintained at 4 °C for 6 h in a growth chamber), and salt stress (plants were irrigated with 200 mM NaCl solution for 6 h). For each stress treatment, mature leaves were collected at the end of the stress period, with three biological replicates harvested per condition. Samples were promptly flash-frozen in liquid nitrogen and stored at −80 °C for subsequent RNA extraction [27].

### 2.2. Identification of POD Genes in Populus simonii

To identify members of the *POD* gene family in the *P. simonii* genome, the complete protein sequences of POD from *A. thaliana* were retrieved from the TAIR database (https://www.arabidopsis.org/, accessed on 9 June 2024). Furthermore, a high-quality chromosome-level reference genome of *P. simonii* was assembled for subsequent analyses, achieving a contig N50 of 24 Mb and a BUSCO complete gene percentage of 98.9%. Subsequently, the PsPOD protein sequences were identified by performing a BlastP search against the *P. simonii* genome data file using TBtools (version 2.145) [28]. Candidate sequences obtained from this analysis were further validated for the presence of conserved POD protein domains using the Conserved Domain Database (CDD) of NCBI (https://www.ncbi.nlm.nih.gov/Structure/bwrpsb/bwrpsb.cgi, accessed on 10 June 2024) and Pfam (http://pfam-legacy.xfam.org/, accessed on 10 June 2024). The integrity of these domains was confirmed, resulting in the identification of candidate *PsPOD* genes. Furthermore, the physicochemical properties of each PsPOD protein, including amino acid length, isoelectric point, molecular weight, and instability index, were analyzed using TBtools software [29]. To investigate the subcellular localization characteristics of *POD* gene family members, this study employed the Wolf PSORT online tool (https://wolfpsort.hgc.jp/, accessed on 30 April 2025) for predictive analysis.

### 2.3. Phylogenetic Analyses of POD Genes in P. simonii

The Muscle algorithm within the MEGA (11.0.13) software package was employed to carry out multiple sequence alignments of the POD proteins from *A. thaliana* and *P. simonii* [30]. Subsequently, a phylogenetic tree depicting the relationships among POD proteins was constructed utilizing the Neighbor-Joining method. This tree was validated through Bootstrap analysis with 1000 replicates, while other parameters were maintained at their default settings. The online platform Interactive Tree of Life (iTOL) (https://itol.embl.de/, accessed on 23 June 2024) was subsequently utilized to refine and annotate the phylogenetic tree of the *PsPOD* family.

### 2.4. Gene Structure and Conserved Motif of the PsPOD Genes

The visualization of gene structures was performed using the “Gene Structure View” module in TBtools (version 2.145), which enabled the depiction of exon–intron organization for the *PsPOD* genes. This process utilized genomic annotation data (GFF format) in combination with gene ID information. To investigate conserved motif distributions in the PsPOD-encoded proteins, the online tool Multiple Em for Motif Elicitation (MEME, version 5.5.3; https://meme-suite.org/meme/tools/meme, accessed on 3 July 2024) was employed. The MEME analysis was conducted using the following parameters: the number of motifs was limited to 10, and the maximum motif width was set to 50 amino acids. All gene structure and motif pattern visualizations were generated using TBtools (version 2.145).

### 2.5. Analysis of the Cis-Acting Elements for the Promoters of the PsPOD Genes

The promoter sequences of the *PsPOD* genes were extracted from the *P. simonii* genome, comprising 2000 base pairs upstream of the translational start codon (ATG). The prediction of cis-acting regulatory elements within these promoter regions was performed using the PlantCARE database (https://bioinformatics.psb.ugent.be/webtools/plantcare/html/, accessed on 18 July 2024) [31]. Visualization of the identified cis-elements and their positional distribution within the promoter regions was carried out using TBtools (version 2.145).

### 2.6. Chromosomal Localization and Synteny Analysis of PsPOD Genes

The chromosomal distribution of the *PsPOD* genes was determined using TBtools (version 2.145), which enabled accurate identification of their positions on the *P. simonii* chromosomes. Based on their physical locations, the *PsPOD* genes were systematically renamed from *PsPOD1* to *PsPOD69*. For comparative genomic analysis, two model plant species, *A. thaliana* and *P. trichocarpa*, were selected to conduct collinearity analysis. This analysis, along with the corresponding visualizations, was performed using the “One-step MCScanX” function in TBtools (version 2.145).

### 2.7. Expression Analysis and Interaction Network Construction of PsPOD Genes

Total RNA was extracted from various organs and tissues subjected to different treatments using the Plant Total RNA Extraction Kit (Biotopped, Beijing, China), following the manufacturer’s instructions. The concentration and integrity of RNA samples were assessed using the Agilent 2100 Bioanalyzer (Santa Clara, CA, USA). High-quality RNA was then used for cDNA library construction and sequencing, which was carried out on the Illumina NovaSeq6000 platform by Wuhan Maiwei Metabolomics Biotechnology Co., Ltd. (Wuhan, China). Raw sequencing reads were processed using Fastp (version 0.20.1) with default parameters to remove low-quality reads and adapter sequences [32]. Clean reads were aligned to the *P. simonii* reference genome using HISAT2 (version 2.2.1) with default settings [33]. Gene expression levels were quantified in FPKM (fragments per kilobase of transcript per million mapped reads) using FeatureCounts (version 2.6.0) [34]. The expression profiles of *PsPOD* genes, derived from RNA-seq data, were visualized as a heatmap using TBtools (version 2.145).

To further explore the expression patterns of *PsPOD* genes under abiotic stress conditions, raw RNA-seq datasets corresponding to heat, cold, and salt stress treatments were retrieved from the NCBI Sequence Read Archive (SRA) under accession numbers SRS1866875, SRR7686816, and SRS1890707. The sequences were aligned to the *P. simonii* reference genome using the same approach, followed by stringent quality control, normalization, and quantification. A count matrix was generated, and the expression levels of *PsPODs* were calculated using FPKM normalization. The resulting data were visualized as a clustered heatmap using TBtools (version 2.145).

Functional prediction of the 69 *PsPOD* protein sequences was performed using the STRING database (https://www.string-db.org/, accessed on 3 August 2024), and a protein–protein interaction (PPI) network was subsequently constructed. The resulting network was visualized and refined using Cytoscape software 2.8 [35].

### 2.8. Quantitative Reverse Transcription Polymerase Chain Reaction (qRT-PCR) Analysis

Total RNA was extracted from the samples using the Plant Total RNA Extraction Kit (Biotopped, Beijing, China), following the manufacturer’s instructions. First-strand cDNA synthesis was carried out using the PrimeScript RT Master Mix (Takara, Osaka, Japan). To validate the RNA-seq expression profiles, twelve *PsPOD* genes were selected for qRT-PCR analysis using gene-specific primers (Table S1), which were designed with TBtools. *Actin* was used as the internal reference gene [36]. qRT-PCR was performed on the CFX Opus 96 system (Bio-Rad, Hercules, CA, USA) with the following thermal cycling conditions: 94 °C for 30 s; 39 cycles of 94 °C for 5 s and 60 °C for 35 s; followed by 95 °C for 15 s, 60 °C for 1 min, and 95 °C for 15 s. Relative gene expression levels were calculated using the 2^−ΔΔCt^ method, with three biological replicates included for each sample [37].

## 3. Results

### 3.1. Identification of PsPOD Genes

As a benchmark, *POD* coding sequences in *A. thaliana* were employed and we used TBtools for alignment and screening combined with the Conserved Domain Database (CDD) tool to detect conserved domains. Through this process, we ultimately identified 69 *POD* family genes in *P. simonii*. Subsequently, the 69 identified *PsPOD* genes were named *PsPOD1* to *PsPOD69* according to their order on homologous chromosomes. We delved into the basic parameters of these *POD* genes to gain a deeper comprehension, including protein length, predicted isoelectric point (pI), molecular weight (MW), and other essential data, as shown in Table S2. The results indicate that the longest POD protein (*PsPOD10*) contains 464 amino acid residues, while the shortest one (*PsPOD6*) has 243 amino acid residues, with an average of 335. The predicted isoelectric points (pI) are in the range of 4.38~9.57, among which 38 POD proteins have a pI greater than 7, accounting for 55.07%. This suggests that most of the POD protein family in *P. simonii* is rich in basic amino acids. The molecular weights (MWs) vary in the range of 26.266~50.397 kDa, and the instability index is in the range of 28.06~58.59. Specifically, 61 proteins are hydrophilic, while 8 proteins are not. In addition, subcellular locations of these *PsPODs* are mainly in the chloroplast, extracellular, vacuole, and cytoplasm (Table S2).

### 3.2. Phylogenetic Analysis of PsPOD Genes

**Figure 1:**
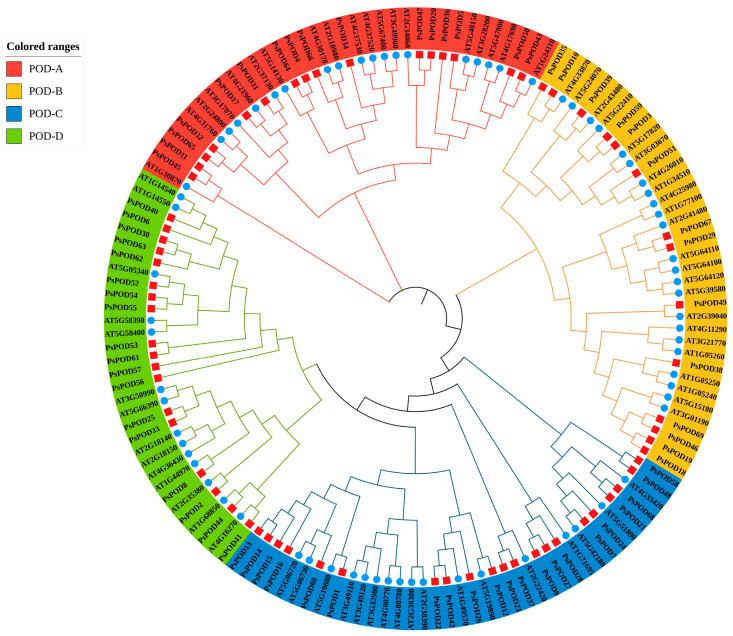
Evolutionary analysis of *Populus simonii* and *Arabidopsis thaliana POD* family members. The phylogenetic tree of PsPOD proteins is divided into four subfamilies, namely POD-A, POD-B, POD-C, and POD-D, represented by red, yellow, blue, and green arcs, respectively. The blue circles represent members of the *POD* gene family from *A. thaliana*, while the red squares represent members of the *POD* gene family from *P. simonii*.

To investigate the evolutionary relationships within the PsPOD protein family of *P. simonii*, a phylogenetic tree was constructed using MEGA11 software with the Maximum Likelihood (ML) method. The analysis was based on the amino acid sequences of 69 *PsPOD* proteins from *P. simonii* and 73 POD proteins from *A. thaliana*, resulting in a total of 142 sequences (Figure 1). This phylogenetic analysis provided insights into the evolutionary divergence and potential functional differentiation among the POD proteins. Based on the tree topology, the *POD* family members were classified into four distinct subfamilies: POD-A (35 members), POD-B (37 members), POD-C (38 members), and POD-D (33 members). Among them, the POD-C subfamily contained the highest number of members, with 21 from *P. simonii* and 17 from *A. thaliana*. All four subfamilies included members from both species, suggesting that diversification of the *POD* gene family predates the divergence of *P. simonii* and *A. thaliana*.

### 3.3. Motif Composition, Protein Conserved Domain and Gene Structure of PsPOD Genes

**Figure 2:**
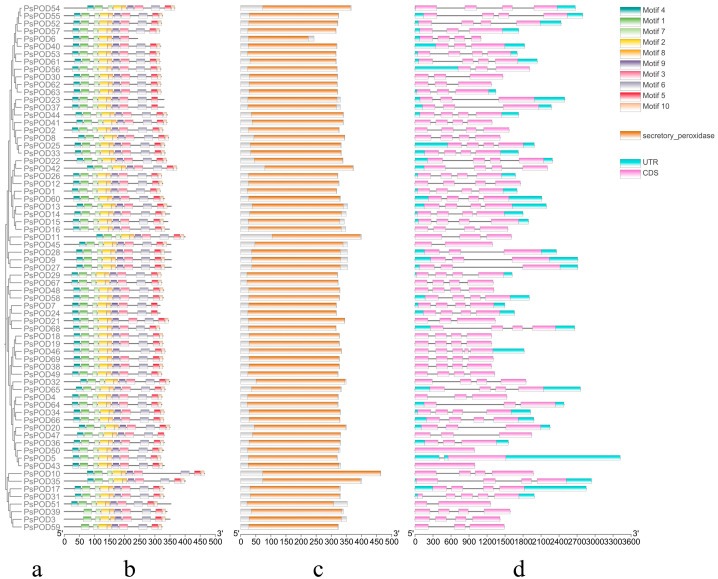
Analysis of conserved domains, motifs and gene structure of *PsPOD* genes. (**a**) Phylogenetic tree of the *PsPOD* gene family. (**b**) Motif composition of PsPOD proteins. Different colored rectangles represent different conserved motifs. (**c**) Conserved domains of *POD* genes in *P. simonii*, marked with orange squares. (**d**) Exon–intron structure of *PsPOD* genes. The UTR regions, exons, and introns are represented by blue squares, pink squares, and black lines, respectively. The scale at the bottom indicates the length of exon and intron segments.

Conservative motif analysis showed that different *PsPOD* family members contained different motifs, ranging from 7 to 10. Except for *PsPOD3*, *6*, *39*, and *59*, which have fewer than 10 motifs, all other genes contain 10 motifs. Among them, *PsPOD6* has the fewest motifs, with only 7, lacking motifs 5, 6, and 10. *PsPOD3*, *39*, and *59* do not contain motif 1 (Figure 2a,b). These findings suggest diversity in the structural and functional properties of the *PsPOD* family members. Protein conserved domain analysis indicated that all members of the *PsPOD* family contain only the plant peroxidase conserved domain (Figure 2c). This finding suggests a common functional core among these proteins related to their peroxidase activity.

Structural analysis revealed that all *PsPOD* genes contain exons, with the number of exons ranging from 1 to 5, and the majority of genes harboring 4 exons. Intron counts varied from 0 to 4, with most genes containing 3 introns. Notably, *PsPOD31* and *PsPOD46* exhibited the highest intron number (four), while *PsPOD5*, *PsPOD45*, and *PsPOD51* each contained only a single intron. *PsPOD43* and *PsPOD50* were identified as intronless genes, composed entirely of exonic sequences (Figure 2d). These structural characteristics indicate a relatively conserved gene architecture across the *PsPOD* family, which may have functional implications in terms of transcriptional regulation and evolutionary diversification.

### 3.4. Analysis of Cis-Elements in PsPOD Gene Promoters

**Figure 3:**
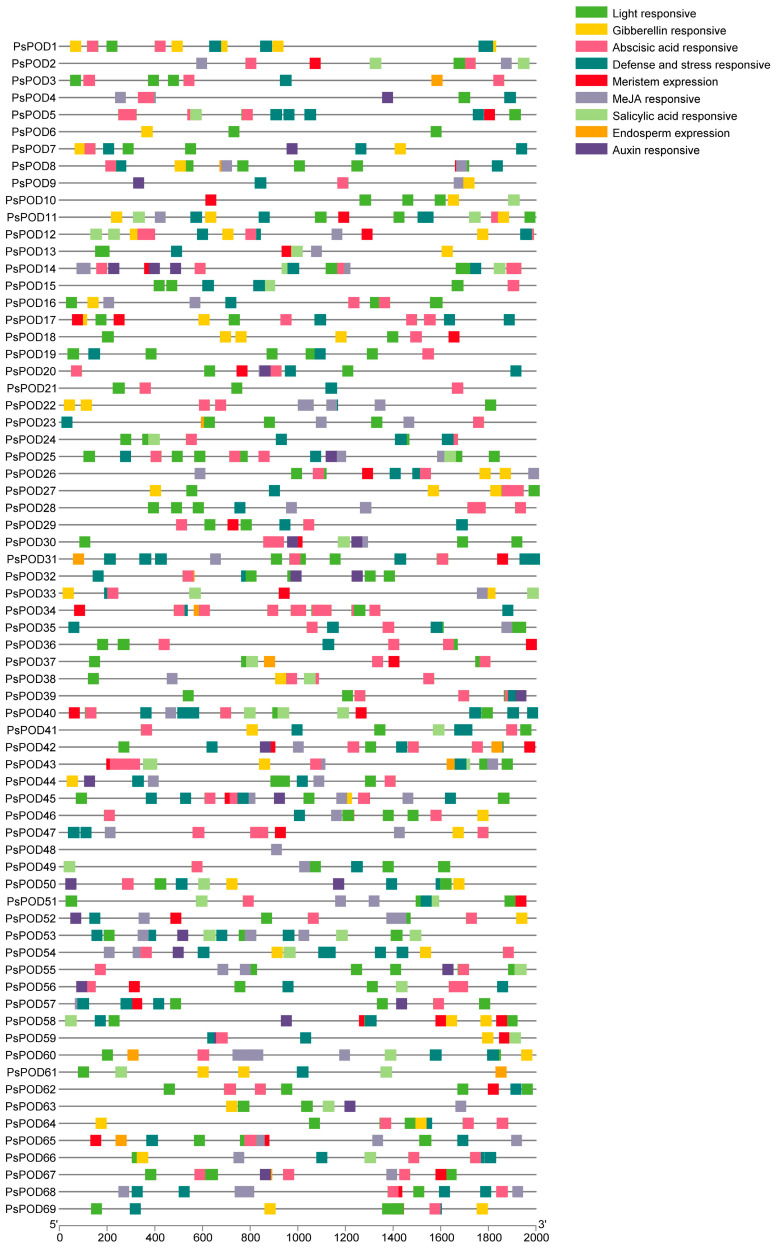
Analysis of the cis-acting elements in the promoter region of the *PsPOD* genes. Each colored box on the right denotes a cis-acting element with a specific function.

To gain further insights into the regulatory mechanisms of the *PsPOD* gene family, cis-acting elements within their promoter regions were analyzed using the PlantCARE online database. All 69 *PsPOD* genes had response elements found in their promoter regions, though the quantity and kind of these elements varied between them (Figure 3). We identified nine categories of cis-acting elements in the promoters of *PsPOD* genes, with the most prevalent being light-responsive elements, totaling 329 instances. Additionally, five types of hormone-responsive elements were predicted, including 170 abscisic acid-responsive, 28 auxin-responsive, 61 gibberellin-responsive, 166 methyl jasmonate-responsive, and 43 salicylic acid-responsive elements. Two kinds of growth and development-related response components were identified, with 13 endosperm expression-related and 43 meristem expression-related elements. Furthermore, 129 defense and stress-responsive elements were also predicted. The data point to *PsPOD* genes being affected by a variety of elements, such as light, growth and development, hormones, and stress; these response components may be directly involved in the expression and control of *PsPOD* genes during developmental stages and in varying stress scenarios.

### 3.5. Chromosomal Location and Synteny Relationship of PsPOD Genes

**Figure 4:**
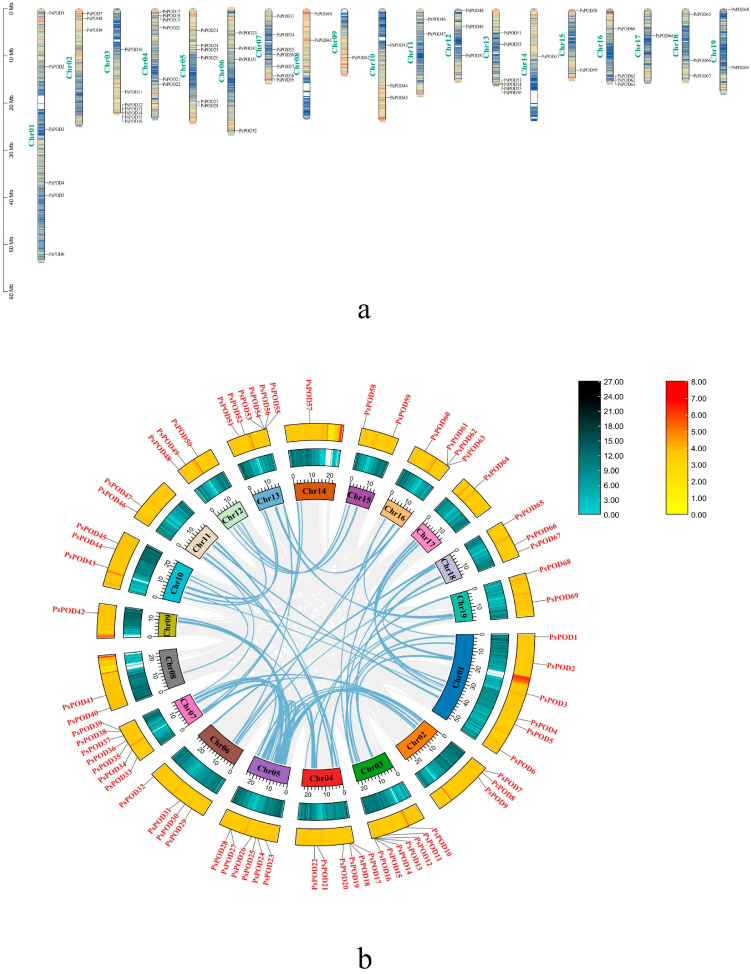
Chromosomal localization and gene collinearity of the *PsPOD* genes. (**a**) Chromosomal localization of genes, the colors within the chromosome represent gene density, with a gradient from yellow to blue signifying a transition from high to low gene density. (**b**) Collinearity analysis of genes, where the numbers on each chromosome box represent the sequence length in bases. The blue–black histogram shows the distribution of gene density, while the red–yellow histogram displays the distribution of GC ratios along the chromosome. The gray lines indicate collinear blocks within the *P. simonii* genome, and the blue lines represent collinear relationships between *POD* regions.

Based on the GFF annotation file of *P. simonii*, all 69 *PsPOD* genes were mapped onto the 19 chromosomes, enabling a comprehensive visualization of their genomic distribution (Figure 4a). Among them, the highest number of genes, seven, are located on the Chr3 and Chr7 chromosomes. The next highest concentration is found on Chr1, Chr4, Chr5, and Chr13, with six members each. The fewest number of genes, only one, are present on Chr9, Chr14, and Chr17. Additionally, gene clusters formed by multiple genes are observed on Chr3, Chr4, Chr5, Chr13, and Chr16.

One of the primary catalysts for genome development is the duplication of genes, including segmental and tandem duplication [38]. In this study, we observed tandem duplication phenomena, as illustrated in Figure 4b. Typically, tandem gene duplication is a fundamental reason for the formation of gene clusters. In our investigation, we observed that some *PsPODs* are located adjacent to each other (Figure 4a). For instance, *PsPOD12–16* on chromosome 3, *PsPOD53*–*56* on chromosome 13, and *PsPOD61–63* on chromosome 16 are all arranged in tandem, suggesting the possibility of tandem duplication relationships among these *PsPODs*. Tandem duplication’s prominent part in the enlargement of the *PsPOD* gene family is evidenced by the results.

Collinearity analysis was performed to investigate the evolutionary relationships among all *PsPOD* genes, using the Circos visualization module integrated in TBtools (Figure 4b). The collinearity analysis identified 44 homologous gene pairs among the 69 *PsPOD* genes, indicating substantial evolutionary relationships within the gene family. Chromosomes 1 and 5 exhibited the highest number of collinear gene pairs, each containing 11 pairs. Notably, several *PsPOD* genes displayed collinear relationships with multiple genes located on different chromosomes, suggesting possible gene duplication events. These findings provide insights into the complex evolutionary dynamics and potential functional redundancy within the *PsPOD* gene family.

### 3.6. Synteny Relationships of PsPOD Genes in P. simonii and Different Species

**Figure 5:**
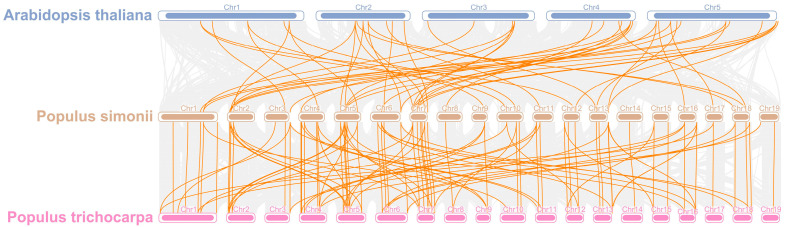
Collinearity analysis of *POD* genes in *P. simonii, A. thaliana* and *Populus trichocarpa*. The gray lines depict collinear segments of homologous plant genomes in *P. simonii*, while the yellow lines represent *POD* gene pairs.

To elucidate the evolutionary trajectory of the *PsPOD* gene family, a comparative synteny analysis was conducted among *P. simonii*, *P. trichocarpa*, and *A. thaliana* (Figure 5). A total of 48 and 98 homologous gene pairs were identified between the 69 *PsPOD* genes of *P. simonii* and the genomes of *A. thaliana* and *P. trichocarpa*, respectively. Notably, chromosome 7 of *P. simonii* exhibited the highest density of orthologous gene pairs, with nine homologs identified with *A. thaliana* and nine with *P. trichocarpa*. In addition, chromosomes 2, 5, and 6 of *P. simonii* displayed high concentrations of both intra- and inter-species syntenic gene pairs, indicating conserved genomic regions. As expected, the degree of homology between *P. simonii* and *P. trichocarpa* was greater than that with *A. thaliana*, reflecting their closer evolutionary relationship.

### 3.7. Regulatory Network and Expression Patterns of PsPOD Genes

**Figure 6:**
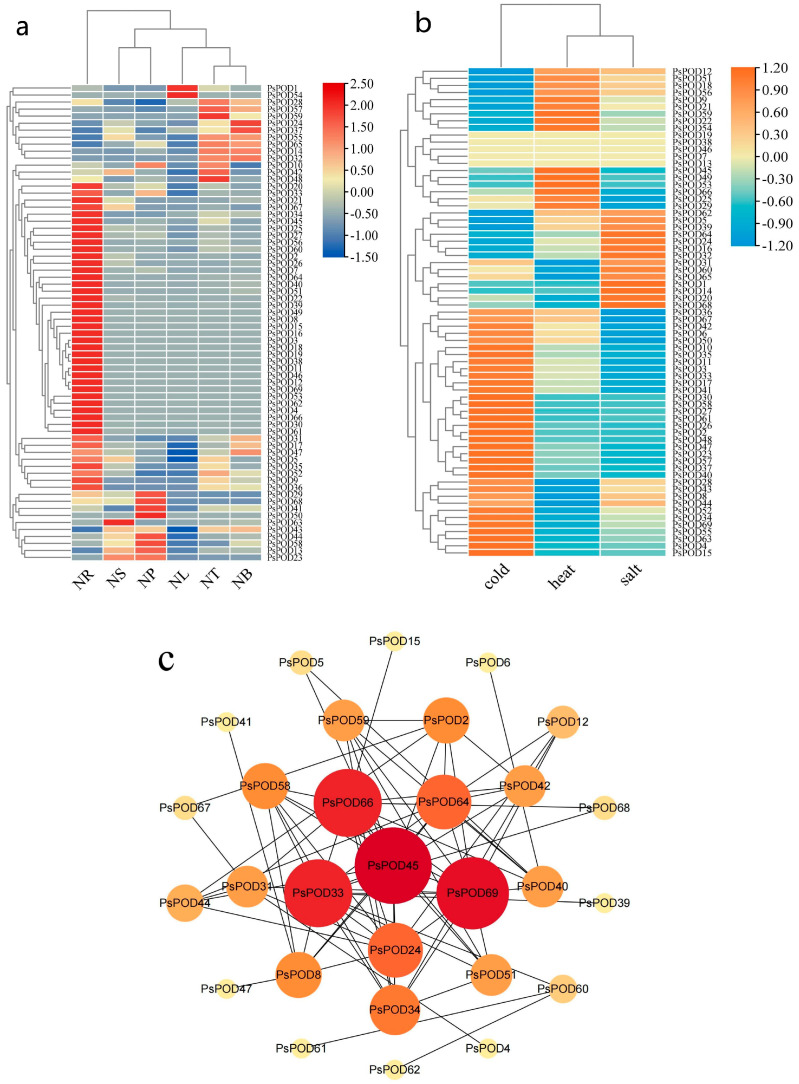
Heatmap and interaction network of expression of *PsPOD* gene family. (**a**) Heatmap of organ/tissue expression levels of the *PsPOD* gene family in *P. simonii*. The color spectrum from red to deep blue indicates gene expression levels from low to high. NR represents roots, NS represents stems, NP represents phloem, NL represents leaves, NT represents terminal buds, and NB represents axillary buds. (**b**) Heatmap of expression levels of the *PsPOD* gene family in *P. simonii* under stress conditions. The color spectrum from orange to sky blue indicates gene expression levels from high to low. Heat represents heat stress, cold represents cold stress, and salt represents salt stress. (**c**) Interaction network of *PsPOD* genes in *P. simonii*. The network contains 29 nodes and 78 edges (interaction combinations). Network nodes symbolize proteins, and edges represent interactions between proteins in the network. The size and color of the circles representing genes indicate their weight value, with larger circles and redder colors reflecting higher weight values.

To investigate the role of the *PsPOD* genes in *P. simonii*, we utilized transcriptome sequencing technology to explore the expression patterns of genes in different organs or tissues of the plant, including roots (NR), stems (NS), phloem (NP), leaves (NL), apical buds (NT), and axillary buds (NB). The results, as shown in Figure 6a, indicate significant expression differences of the *PsPOD* gene across various organs/tissues of *P. simonii*. For instance, most *PsPOD* genes show higher expression levels in roots compared to other organs/tissues. However, *PsPOD63* exhibits higher expression in the stem than in other tissues, while *PsPOD50* demonstrates elevated expression in the phloem. Additionally, *PsPOD1* and *PsPOD54* display higher expression in leaves, and *PsPOD59* has increased expression in apical buds compared to other tissues.

Given the discovery of cis-acting elements responsible for stress response in the promoter of *PsPOD* genes, this study utilized RNA-seq data from *P. simonii* leaves under heat, cold, and salt stress conditions to explore the expression pattern of *PsPOD* genes under stress conditions (Figure 6b). Under cold stress, half of the *PsPOD* genes exhibited higher expression levels. Conversely, under heat and salt stress, the majority of *PsPOD* genes showed a reduction in expression. Notably, a small subset of genes displayed significant alterations in expression levels, manifesting as increased gene expression under various stress conditions.

Moreover, we constructed an interaction network comprising all *PsPOD* genes, utilizing the STRING database and Cytoscape software, to further explore the potential links between them (Figure 6c). The results indicate that among the 69 *PsPOD* genes, 29 genes are involved in constructing a complex gene regulatory network, and some of these genes often act as hub genes in the regulatory network. For instance, *PsPOD45* interacts with 14 other genes as a hub gene, while *PsPOD69* interacts with 13 other genes. The identification of this regulatory network provides valuable information for a better understanding of the role of *POD* genes in development and stress responses.

### 3.8. qRT-PCR Analysis

**Figure 7:**
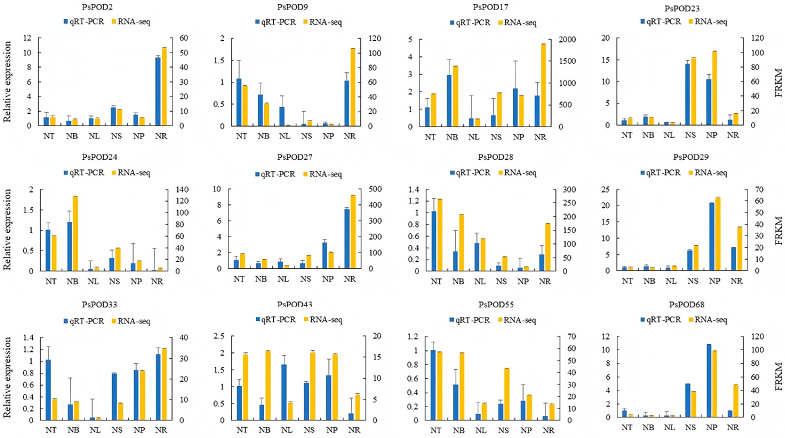
Gene expression profiles of RNA-seq and qPCR of the *PsPOD* gene family. The x-axis represents six different organs or tissues (abbreviations are shown in Figure 6). The left y-axis shows the expression data from qRT-PCR. The right y-axis indicates the relative expression levels of genes validated by RNA-seq. Error bars represent standard errors.

To further investigate the organ- or tissue-specific expression patterns of *PsPOD* genes, twelve genes were randomly selected for qRT-PCR analysis using gene-specific primers. The qRT-PCR results were consistent with the RNA-seq data, thereby validating the reliability of the transcriptomic analysis in reflecting *PsPOD* gene expression levels (Figure 7).

## 4. Discussion

Class III peroxidases play essential roles in plant growth, development, and responses to both biotic and abiotic stresses [39]. Although comprehensive genome-wide analyses of the *POD* gene family have been conducted in several plant species, including *A. thaliana* [18], rice [22], *P. trichocarpa* [20], and maize [22], a systematic characterization of this gene family in *P*. *simonii* has not yet been reported. In the present study, we performed a genome-wide identification and analysis of the *POD* gene family in *P. simonii*. The expansion, structure, and diversity of gene families are often shaped by complex evolutionary processes, including genome duplication and environmental adaptation. A total of 69 *PsPOD* genes were identified in *P. simonii*. This number is comparable to that reported in sweet orange (73) [40], *A. thaliana* (73) [18], carrot (75) [41], and lychee (77) [24], but lower than in cassava (91) [23], Chinese pear (94) [25], and maize (119) [22]. Conversely, it is higher than the *POD* gene count in sesame (45) [42]. These differences in gene family size among species likely reflect lineage-specific evolutionary trajectories driven by genomic duplications and adaptive pressures.

Based on chromosomal localization analysis, *PsPOD* genes were identified on all chromosomes of *P. simonii*. Among them, a higher number of *PsPOD* genes were found on chromosomes 3 and 7, while fewer genes were detected on chromosomes 9, 14, and 17. This indicates that there is a phenomenon of uneven distribution of genes during the evolutionary process. In addition, the results indicated the existence of repeated sequences of *PsPOD* genes arranged in tandem across several chromosomes of *P. simonii*, constituting the primary mechanism for the enlargement of the *PsPOD* gene family. The expansion of the *POD* gene family in *P. simonii* is notably affected by tandem repetitive sequences, which is consistent with the results observed in studies focused on the *POD* gene family in *P. trichocarpa* [20]. In contrast to the results of this study, the *POD* gene family of pear was found to have been primarily augmented by segmental duplication [25]. Notably, both segmental duplication and tandem duplication were found to be influential in the expansion of the *POD* gene family of corn [22]. These findings reveal notable variations in the patterns of *POD* gene amplification among *P. simonii*, corn, and Chinese pear, indicating that the expansion of the *POD* gene family differs across species.

We conducted a synteny analysis between *P. simonii*, *A. thaliana*, and *P. trichocarpa POD* genes. The findings revealed that *P. simonii* shares 98 pairs of homologous genes with *P. trichocarpa*, which is 50 pairs more than its homologous genes with *A. thaliana*. Compared to *A. thaliana*, the *POD* genes of *P. simonii* and *P. trichocarpa* exhibit higher homology. This may be attributed to their closer phylogenetic relationship, as both belong to the same genus. Conversely, their more distant relationship to *Arabidopsis*, which is a herbaceous plant while the former two are woody plants, could account for the lower homology. Through analysis of gene structure and conserved motifs, it was found that all members of the *POD* gene family in *P. simonii* contain only one typical peroxidase domain. Furthermore, the majority of these members possess all ten motifs (65/69), suggesting their potential role in maintaining intracellular ROS balance. The diverse protein motifs likely determine their involvement in different regulatory pathways and the execution of distinct biological functions [43].

It is widely believed that cis-regulatory elements are crucial in gene expression regulation by acting as binding sites for transcription factors [44]. In this study, we identified a variety of cis-regulatory elements in the promoter regions of *PsPOD* genes, including motifs responsive to light, hormones, defense, and stress. The presence of light- and hormone-responsive elements such as G-box, TGA-element, and ABRE is consistent with canonical regulatory motifs previously characterized in *A. thaliana* and *Oryza sativa POD* genes [11,18], indicating conserved transcriptional control mechanisms in plant responses to abiotic stimuli. Additionally, the enrichment of defense- and stress-responsive elements agrees with earlier observations in *P. trichocarpa* defense-related genes [45], suggesting a conserved role of *POD* genes in biotic stress responses within the *Populus* genus. Importantly, we also observed a notable distribution of meristem-specific elements in the promoter regions of *PsPOD* genes. To our knowledge, such elements have not been systematically reported in *POD* genes of Solanaceae species, including *Capsicum annuum* and *Nicotiana tabacum* [46,47], indicating a potential Salicaceae-specific regulatory feature. This may reflect a functional adaptation associated with the regulation of secondary growth and cambial development, processes that are particularly prominent in woody perennials like *Populus*. Overall, the presence of both conserved and potentially lineage-specific cis-elements suggests that while core *POD* gene regulation is maintained across plant species, additional regulatory complexity may have evolved in *Populus* to support its perennial growth habit and stress adaptability.

Gene expression at the RNA level is a key determinant of biological phenotypes [48]. In this study, we found that most *PsPOD* genes exhibited higher expression levels in root tissues, a pattern consistent with findings in *A. thaliana* [18] and maize [22]. In *A. thaliana*, POD genes are co-expressed with those encoding root hair-specific proteins and nitrate transporters, while in maize, they show coordinated expression with genes involved in root cell wall biosynthesis and mycorrhizal symbiosis. These conserved co-expression patterns across diverse species strongly support the critical role of POD genes in root-related physiological processes. Furthermore, the elevated expression of *PsPOD* genes in roots suggests their potential involvement in maintaining root functionality and enhancing plant adaptation to environmental stresses. Compared to other tissues, the overall expression levels of *PsPOD* genes in the leaves of *P. simonii* were relatively low; however, two genes, *PsPOD1* and *PsPOD54*, exhibited notably high expression. This may indicate that these genes play specific roles in leaf function or development, potentially compensating for the reduced activity of other *PsPOD* genes during later developmental stages [49,50,51]. Different functional genes may be expressed in roots and leaves, leading to distinct physiological and biochemical characteristics in these tissues.

Abiotic stresses, such as salinity and temperature extremes, are major limiting factors for plant growth and development. To survive under such conditions, plants activate complex regulatory networks at morphological, physiological, and transcriptional levels, including the upregulation of antioxidant defense genes like *PODs* [52]. In this study, expression profiling under multiple abiotic stress conditions demonstrated that *PsPOD* genes are actively involved in stress responses, as reflected by substantial changes in their transcript levels. Among the tested stresses, cold stress elicited the most prominent transcriptional activation, with *PsPOD2*, *4*, *15*, *26*, *27*, *30*, *37*, *48*, *58*, and *61* showing markedly elevated expression. In contrast, *PsPOD45*, *49*, and *53* were specifically responsive to heat stress, while only *PsPOD1* and *14* exhibited significant induction under salt stress. Notably, no individual gene exhibited strong responsiveness across all three abiotic stresses, indicating that *PsPOD* gene responses are stress-type specific. Similar patterns have been observed in other plant species. In passion fruit (*Passiflora edulis*), several *PePOD* genes—including *PePOD1*, *PePOD3*, *PePOD22*, *PePOD26*, and *PePOD29*—were significantly induced by cold treatment [53]. In cassava (*Manihot esculenta*), *MePOD13* and *MePOD16* were consistently upregulated under drought stress across multiple genotypes [23]. Likewise, in grapevine (*Vitis vinifera*), approximately 52% of *VvPOD* genes were upregulated under salt stress, while drought stress led to the downregulation of 72% of family members [54]. These comparative findings support the stress-responsive nature of *POD* genes across diverse plant taxa, while also highlighting species-specific and stress-type-specific regulatory patterns. The observed expression divergence of *PsPOD* genes in *P. simonii* may reflect lineage-specific regulatory adaptations to temperature-related stresses, consistent with the ecological distribution of the species. Additionally, the RNA-seq results were validated by qRT-PCR analysis of *PsPOD* gene expression across six organs and tissues. The qRT-PCR expression patterns were largely consistent with the transcriptome data, thereby confirming the reliability of RNA-seq in evaluating *PsPOD* gene expression profiles.

Protein–protein interactions are fundamental to the execution of biological functions within living systems [55]. In the present study, protein interaction network analysis revealed that only 29 *PsPOD* proteins were involved in detectable interactions. Among them, *PsPOD45*, *PsPOD69*, *PsPOD33*, and *PsPOD64* were identified as major hub genes, each interacting with 14, 13, 12, and 12 other proteins, respectively. These hub genes are likely involved in a wide range of biological processes, including signal transduction, transcriptional regulation, energy metabolism, metabolite processing, and cell cycle control. The broader interaction network consists of multiple genes and proteins whose coordinated functions collectively influence plant growth and development under varying environmental conditions. Given that the specific roles of most *PsPOD* genes remain poorly understood, the identification of these four central genes provides valuable targets for future functional studies aimed at elucidating protein mechanisms and regulatory networks in *P. simonii*.

## 5. Conclusions

In this study, a total of 69 class III peroxidase (*PsPOD*) genes were identified in the *P. simonii* genome through genome-wide analysis. These genes are unevenly distributed across all 19 chromosomes. Based on phylogenetic relationships, gene structures, and conserved motif compositions, the *PsPOD* genes were classified into four distinct subfamilies, with members of each subfamily exhibiting similar exon–intron organization and motif arrangements. All *PsPOD* proteins were found to contain a single conserved domain characteristic of the *POD* family. Promoter analysis revealed the presence of multiple cis-acting elements related to light responsiveness, hormone signaling, growth and development, and abiotic stress responses, suggesting the regulatory complexity of this gene family. Expression profiling across different tissues and stress conditions demonstrated that *PsPOD* genes exhibit diverse and tissue-specific expression patterns. Furthermore, four hub genes were identified from the protein–protein interaction network, providing potential candidates for future functional characterization. Overall, this study presents the first comprehensive investigation of the *PsPOD* gene family in *P. simonii* and lays a solid foundation for further studies on their biological roles and stress response mechanisms.

## Data Availability

All data generated or analyzed during this study are included in this published article.

## Supplementary Materials

The following supporting information can be downloaded at https://www.mdpi.com/article/10.3390/antiox14050602/s1: Table S1: Specific primers used for qRT-PCR analysis; Table S2: Basic information of the *POD* genes in *Populus simonii*.
